# Impact of Previous Nephrectomy on Clinical Outcome of Metastatic Renal Carcinoma Treated With Immune-Oncology: A Real-World Study on Behalf of Meet-URO Group (MeetUro-7b)

**DOI:** 10.3389/fonc.2021.682449

**Published:** 2021-06-08

**Authors:** Marco Stellato, Daniele Santini, Elena Verzoni, Ugo De Giorgi, Francesco Pantano, Chiara Casadei, Giuseppe Fornarini, Marco Maruzzo, Andrea Sbrana, Giuseppe Di Lorenzo, Mariella Soraru, Emanuele Naglieri, Sebastiano Buti, Rocco De Vivo, Andrea Napolitano, Francesca Vignani, Claudia Mucciarini, Francesco Grillone, Giandomenico Roviello, Marilena Di Napoli, Giuseppe Procopio

**Affiliations:** ^1^ Department of Medical Oncology, Campus Bio-Medico University of Rome, Rome, Italy; ^2^ Medical Oncology Department, Fondazione IRCCS Istituto Nazionale dei Tumori, Milan, Italy; ^3^ Department of Medical Oncology, IRCCS Istituto Romagnolo per lo Studio dei Tumori (IRST) Dino Amadori, Meldola, Italy; ^4^ Onco-ematological Department, IRCCS Ospedale Policlinico San Martino, Genova, Italy; ^5^ Medical Oncology Unit 1, Department of Oncology, Istituto Oncologico Veneto IOV IRCCS, Padua, Italy; ^6^ Department of Surgical, Medical and Molecular Pathology and Critical Area Medicine, University of Pisa, Pisa, Italy; ^7^ Oncologia, Università del Molise, Campobasso, Italy; ^8^ Oncology Unit, Camposampiero General Hospital, Padova, Italy; ^9^ Division of Medical Oncology, Istituto Tumori Bari Giovanni Paolo II—IRCCS, Bari, Italy; ^10^ Medical Oncology Unit, University Hospital of Parma, Parma, Italy; ^11^ Department of Oncology, San Bortolo General Hospital, Vicenza, Italy; ^12^ AO Ordine Mauriziano, SCDU Oncologia, Turin, Italy; ^13^ U.O. Medicina Oncologica, Ospedale Ramazzini, Carpi-AUSL Modena, Carpi, Italy; ^14^ Azienda Ospedaliero-Universitario “Mater Domini”, Policlinico of Catanzaro, Catanzaro, Italy; ^15^ Department of Health Sciences, University of Florence, Firenze, Italy; ^16^ Department of Urology and Gynecology, Istituto Nazionale Tumori IRCCS Fondazione “G. Pascale”, Napoli, Italy

**Keywords:** nephrectomy, immune-oncology, metastatic renal cell carcinoma, immunotherapy, nivolumab

## Abstract

**Background:**

Immune-Oncology (IO) improves Overall Survival (OS) in metastatic Renal Cell Carcinoma (mRCC). The prognostic impact of previous Cytoreductive Nephrectomy (CN) and radical nephrectomy (RN), with curative intent, in patients treated with IO is not well defined. The aim of our paper is to evaluate the impact of previous nephrectomy on outcome of mRCC patients treated with IO.

**Methods:**

287 eligible patients were retrospectively collected from 16 Italian referral centers adhering to the MeetUro association. Patients treated with IO as second and third line were included, whereas patients treated with IO as first line were excluded. Kaplan–Meier method and log-rank test were performed to compare Progression Free Survival (PFS) and OS between groups. In our analysis, both CN and RN were included. The association between nephrectomy and other variables was analyzed in univariate and multivariate setting using the Cox proportional hazard model.

**Results:**

246/287 (85.7%) patients had nephrectomy before IO treatment. Median PFS in patients who underwent nephrectomy (246/287) was 4.8 months (95%CI 3.9–5.7) vs 3.7 months (95%CI 1.9–5.5) in patients who did not it (HR log rank 0.78; 95%CI 0.53 to 1.15; *p* = 0.186). Median OS in patients who had previous nephrectomy (246/287) was 20.9 months (95%CI 17.6–24.1) *vs* 13 months (95%CI 7.7–18.2) in patients who did not it (HR log rank 0.504; 95%CI 0.337 to 0.755; *p* = 0.001). In the multivariate model, nephrectomy showed a significant association with OS (HR log rank 0.638; 95%CI 0.416 to 0.980), whereas gland metastases were still associated with better outcome in terms of both OS (HR log rank 0.487; 95%CI 0.279 to 0.852) and PFS (HR log rank 0.646; 95%CI 0.435 to 0.958).

**Conclusions:**

IO treatment, in patients who had previously undergone nephrectomy, was associated with a better outcome in terms of OS. Further prospective trials would assess this issue in order to guide clinicians in real word practice.

## Introduction

Kidney cancer represents 5% of estimated new cases of cancer in men and 3% in women, being the 13th most commonly diagnosed solid malignancy (1, 2).

Approximately 20% of patients will develop metastases after nephrectomy, while 15% patients have already developed synchronous metastatic disease at the time of diagnosis (2, 3).

Radical Nephrectomy (RN) and Partial Nephrectomy (PR) with curative intent can be considered standard of care in patients with localized disease (4–7). In the management of metastatic renal cell carcinoma (mRCC) patients, randomized data from the “interferon era” demonstrated that Cytoreductive Nephrectomy (CN) improves survival, decreasing the risk of death (8). Despite the retrospective data from IMDC by Heng et al. and the prospective results from CARMENA and SURTIME trial (9, 10), CN remains controversial in patients treated with VEGF-targeted therapy. Recently, Immune-Oncology (IO), alone or in combination, has changed the standard of care in mRCC due to the high rate of survival in pretreated and treatment-naïve patients (11–13). In CHECKMATE214, Overall Survival (OS) favored nivolumab plus ipilimumab over sunitinib, also in patients who had previous nephrectomy. Updated analysis confirms that median OS was longer among those randomized to nivolumab-ipilimumab with target kidney lesion (14). Nevertheless, in patients treated with IO, the role of previous CN or RN has not been defined. It is unclear if previous nephrectomy affects outcome in patients treated with IO. Data about response of primary renal tumor to IO are partial and prospective trials evaluating the effect of nephrectomy in patients treated with IO are lacking.

Previous reports demonstrated some changes in the immune system after nephrectomy, but data are not conclusive (15). Therefore, the aim of our study was to retrospectively evaluate the impact of previous nephrectomy on mRCC patients treated with IO.

## Patients and Methods

We retrospectively collected data of mRCC patients treated with IO in 16 Italian referral centers adhering to the Meet-Uro group, between February 2017 and January 2020.

Inclusion criteria were at least 18 years old at the time of enrollment, histological diagnosis of RCC and radiological diagnosis of metastatic disease.

Patients treated with IO, as single agent or in combination, were considered eligible. Patients enrolled in the expanded access program of nivolumab or nivolumab–ipilimumab were excluded. Patients treated with first line IO were excluded to homogenize our population, whereas patients treated with IO as second and third line were included.

Baseline characteristics were collected at the start of immunotherapy. Outcome data, including PFS and toxicities, were collected too. Data included site of metastatic disease, duration of first line and subsequent IO therapy, previous CN or RN. Glandular metastasis included metastasis in glandular organs such as thyroid, pancreas and adrenal gland.

The International Metastatic RCC Database Consortium (IMDC) prognostic risk group was computed at the index date based on the presence of six individual risk factors including time from diagnosis to systemic treatment <1 year, hemoglobin < lower limit of normal, calcium >10 mg/dl, platelet > upper limit of normal, neutrophil > upper limit of normal, Performance Status (PS) <80% (Karnofsky) (16).

Primary endpoint was to evaluate difference in IO-OS between patients who previously received nephrectomy and patients who did not it. IO-OS was defined as the time from the start of IO to death.

Secondary endpoints were to evaluate difference in PFS between the twogroups of patients. PFS was defined as the time from the start of IO to radiological or clinical progression.

Patients with no evidence of death were censored at the date of last tumor assessment.

Real-world physician-assessed progression and response was based on clinical criteria or radiographic criteria using Response Evaluation Criteria in Solid Tumors (RECIST) guidelines (17), with imaging assessments occurring at clinically variable time points.

Baseline demographic and clinical characteristics have been described using frequencies and percentages for categorical variables.

Descriptive analysis was made using median values and ranges. Kaplan–Meier method and Mantel–Haenszel log-rank test were performed to compare differences in OS and PFS between groups. The association between nephrectomy and other variables was analyzed in univariate and multivariable setting using the Cox proportional hazard model. Variables to be included in multivariate analysis were selected according to the levels of significance in cox regression univariate analysis. P-values <0.05 were considered significant. All statistical analyses were performed using SPSS software (version 19.00, SPSS, Chicago).

Written informed consent for patient information to be published was provided by the patients or a legally authorized representative. All participating centers received local ethics approval for data collections. The study was conducted in accordance with good clinical practice and the Declaration of Helsinki.

## Results

287 patients were considered eligible. Characteristics of patients are described in **Table 1**. All patients received nivolumab as IO.

**Table 1:** Characteristics of patients.

	N (%)
**Age**	
median	69.4 y
**Sex**	
M	206 (71.7)
F	81 (28.3)
**Nephrectomy**	
Y	246 (85.7)
N	41 (14.3)
**Cytoreductive Nephrectomy**	95 (33.1)
**Clear cell**	
Y	246 (86.0)
N	41 (14.0)
Sarcomatoid	
Y	36 (12.5)
N	251 (87.5)
**IO Line**	
2	195 (68)
3	73 (25.4)
further line	19 (6.6)
**ECOG PS at IO start**	
0	145 (50.5)
1	116 (40.4)
2	26 (9.0)
**Previous TKI treatment**	
sunitinib	178 (62.0)
pazopanib	97 (33.8)
cabozantinib	22 (7.6)
sorafenib	6 (2.0)
everolimus	19 (6.6)
axitinib	36 (12.5)
lenvatinib everolimus	6 (2.0)
tivozanib	4 (1.4)
lenvatinib	1 (0.3)
**Metastatic sites**	
lynphonodes	128 (44.6)
lung	122 (42.5)
bone	84 (29.2)
liver	33 (11.5)
brain	12 (4.2)
gland	37 (12.9)
peritoneum	14 (4.8)
**IMDC score**	
good	82 (28.6)
Intermediate	176 (61.3)
poor	29 (10.1)

M, male; F, female; IO, Immune-Oncology; ECOG PS, Eastern Cooperative Oncology Group Performance status; TKI, tyrosine-Kinase Inhibitor; IMDC, International Metastatic renal cell carcinoma Database Consortium.

246/287 (85.7%) patients had nephrectomy, whereas 41 (14.3%) patients did not it. 95 (33.1%) patients had CN and 151 (52.6%) patients had RN with curative intent. Nephrectomy was performed before IO treatment.

136/287 patients (47.4%) had synchronous metastatic disease, whereas 151/287 patients (52.6%) had metachronous disease.

G3–G4 immune-related Adverse Events (irAEs) were reported in 24/287 patients (8.3%).

At a median follow up of 24.7 months, 114/287 patients (56.4%) received target therapy (TT), such as mTOR inhibitors and VEGFR inhibitors at progression to IO, whereas 68/287 patients (25.4%) did not receive further treatment for clinical deterioration. 68/287 patients (23.7%) continued IO beyond progression.

52/287 patients (18.2%) were still in treatment at the time of analysis.

**Figure 1:**
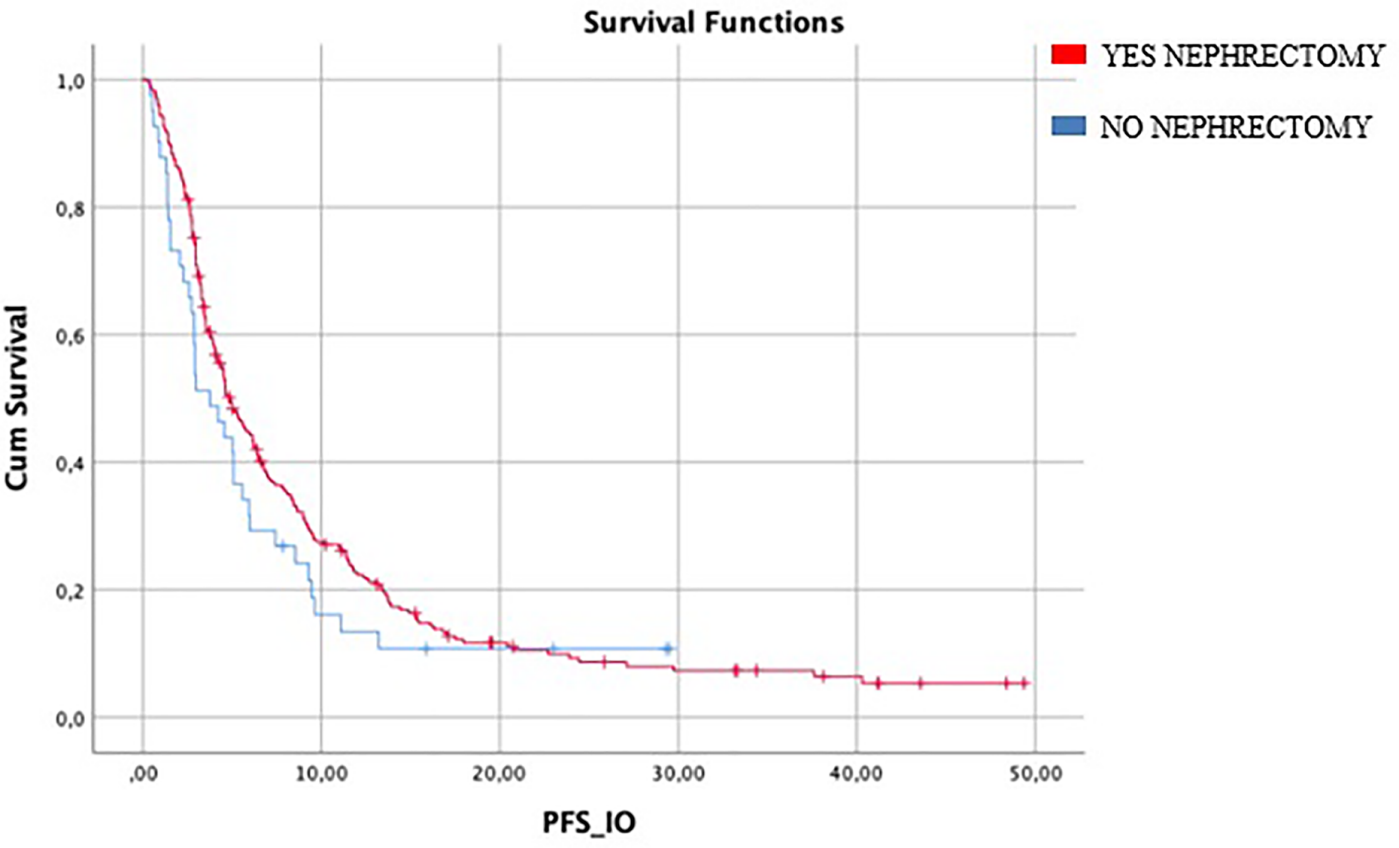
Median mIO-PFS in patients who underwent nephrectomy (246/287) was 4.8 months *vs* 3.7 months in patients who did not (HR log rank 0.78; 95%CI 0.53 to 1.15; *p* = 0.186). mIO-PFS (mPFS in patient treated with IO).

Median IO-PFS was 4.6 months (95%CI 3.85–5.42). Median PFS in patients who underwent nephrectomy (246/287) was 4.8 months (95%CI 3.9–5.7) vs 3.7 months (95%CI 1.9–5.5) in patients who did not it (HR log rank 0.78; 95%CI 0.53 to 1.15; *p* = 0.186) (**Figure 1**) (**Table 2**).

**Table 2:** Median PFS difference between groups of patients treated with IO.

	mPFS	*p* value
**Nephrectomy**		
Y	4.8 (3.9–5.7)	0.186
N	3.7 (1.9–5.5)	
**Histology**		
Clear cell	4.8 (3.9–5.7)	0.829
Non clear cell	4.6 (2.8–6.4)	
**Sarcomatoid variant**		
Y	4.3 (2.0–6.6)	0.97
N	4.8 (3.9–5.7)	
**Bone metastasis**		
Y	4.1 (3.0–5.2)	0.093
N	5.0 (4.0–5.9)	
**Lynphonodes metastasis**		
Y	5.0 (3.4–6.6)	0.216
N	4.6 (3.9–5.3)	
**Lung metastasis**		
Y	5.5 (3.6–6.7)	0.089
N	4.5 (4.2–5.3)	
**Liver metastasis**		
Y	4.6 (0.7–5.3)	0.813
N	5.0 (4.0–6.1)	
**Gland metastasis**		
Y	6.5 (2.8–6.9)	**0.022**
N	4.6 (3.8–5.5)	
**IMDC SCORE**		
0	6.1 (1.0–4.0)	**0.044**
1	4.5 (0.4–3.6)	
2	3.3 (2.1–0.0)	
**ECOG PS**		
0	5.5 (3.8–7.3)	0.25
1	4.5 (3.9–5.1)	
2	3.0 (2.2–3.8)	
**G3–G4 toxicities**		
Y	5.0 (3.2–6.7)	0.9
N	4.8 (3.9–5.7)	

Y, Yes; N, No; IMDC, International Metastatic Renal Cell Carcinoma Database Consortium;

ECOG PS, Eastern Cooperative Oncology Group Performance status; G, Grade; mPFS, Median Progression Free Survival; IO, Immune-Oncology.

Groups of patients with different IMDC score and with gland metastasis had statistically difference in PFS when treated with IO.

Bold values represent statistically significant value.

**Figure 2:**
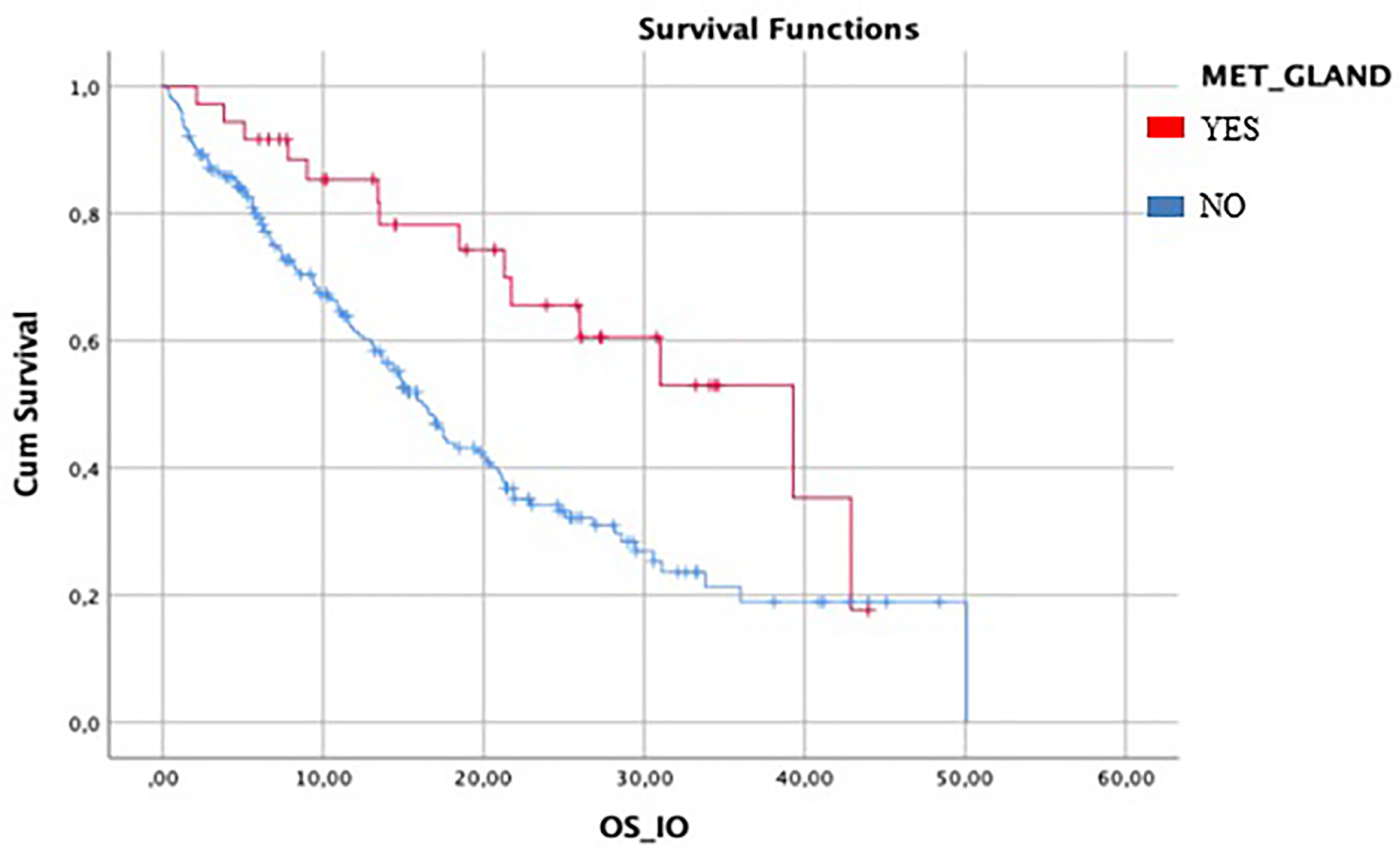
Difference between mIO-OS between patient with gland metastasis and patient without gland metastasis. Median IO-OS was longer in patients with gland metastasis.

Median IO-OS in the entire population was 18.5 months (95%CI 15.5–21.4). In patients with metastasis to glandular organs (37/287), mOS was 39.3 months (95%CI 22.5–43.5) compared to 16.2 months (95%CI 13.9–23.8) in patients without gland metastasis (250/287) (**Figure 2**).

**Figure 3:**
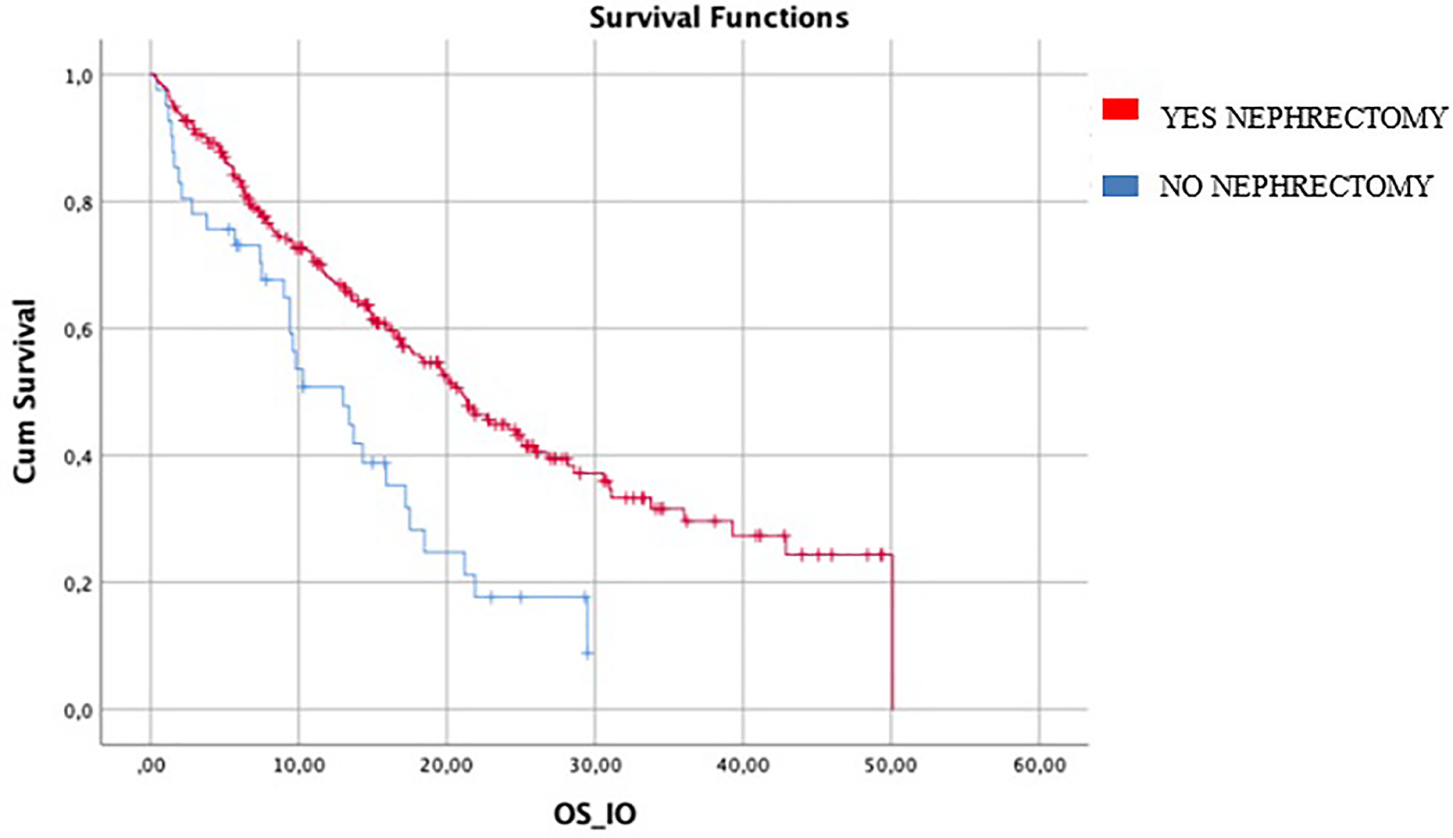
difference in mIO-OS between who underwent nephrectomy was 20.9 (95%CI 17.6–24.1) *vs* 13.0 (95%CI 7.7–18.2) in patients who did not. IO-OS (median OS in patients treated with IO).

Median OS in patients who had previous nephrectomy (246/287) was 20.9 months (95%CI 17.6–24.1) vs 13 months (95%CI 7.7–08.2) in patients who did not it (HR log rank 0.504; 95%CI 0.337 to 0.755; *p* = 0.001) (**Figure 3**).

In patients with synchronous metastatic disease (136/287), mOS was 20.5 months for those who underwent CN, compared to 13 months in patients who did not it (HR log rank 0.51; 95%CI 0.305 to 0.855; *p* = 0.0024). On the other hand, mPFS was 4.6 months in patients who underwent CN vs 3.7 months in patients who did not it (HR log rank 0.83; 95%CI 0.554 to 1.247; *p* = 0.34) (**Table 3**).

**Table 3:** mOS differences between groups of patients treated with IO.

	mIO-OS	*p* value
**Nephrectomy**
Y	20.9 (17.6–24.1)	**0.001**
N	13.0 (7.7–18.2)	
**Histology**
Clear cell	18.5 (15.4–21.5)	0.654
Non-clear cell	17.0 (0.0–38.9)	
**Sarcomatoid component**		
Y	19.7 (11.3–28.0)	0.923
N	18.5 (15.3–21.6)	
**Bone metastasis**		
Y	18.5 (11.5–27.6)	0.814
N	19.5 (16.1–26.1)	
**Lynphonodes metastasis**		
Y	17.5 (14.0–20.9)	0.908
N	20.2 (16.3–24.0)	
**Lung metastasis**		
Y	16.6 (11.6–26.0)	0.576
N	18.5 (14.8–27.1)	
**Liver metastasis**		
Y	16.6 (10.7–24.7)	0.280
N	18.5 (14.9–26.6)	
**Gland metastasis**		
Y	39.3 (22.5–43.5)	**0.004**
N	16.2 (13.9–23.8)	
**IMDC SCORE**		
0	22.9 (15.7–33.3)	**0.004**
1	17.2 (12.3–25.1)	
2	15.3 (8.3–20.2)	
**ECOG PS**		
0	19.7 (15.9–23.4)	0.208
1	20.9 (14.3–27.4)	
2	10.9 (5.6–16.1)	
**IO line**		
2	19.7 (15.9–23.4)	
3	20.5 (14.5–26.4)	
4	11.9 (3.8–19.9)	
5	1.3 (NR-NR)	
**G3–G4 toxicities**		
Y	17.0 (4.7–29.2)	0.761
N	19.7 (16.6–22.7)	

Y, Ye; N, No; mOS, median Overall Survival; IO, Immune-Oncology; IMDC, International

Metastatic Renal Cell Carcinoma Database Consortium; ECOG PS, Eastern Cooperative Oncology Group Performance Status; G, Grade.

Nephrectomy, gland metastasis and IMDC score demonstrated a significant difference in mOS between groups.

Bold values represent statistically significant value.

In the multivariate model, including gland metastasis and IMDC score, nephrectomy showed significant association with OS (HR log rank 0.638; 95%CI 0.416 to 0.980), whereas gland metastases were still associated with better outcome in terms of both OS (HR log rank 0.487; 95%CI 0.279 to 0.852) and PFS (HR log rank 0.646; 95%CI 0.435 to 0.958). However, IMDC score showed significant association with both OS (HR log rank 1.352; 95%CI 1.020 to 1.791) and PFS (HR log rank 1.27; 95%CI 1.016 to 1.587).

## Discussion

IMDC and Memorial Sloan-Kettering Cancer Center (MSKCC) score are currently the gold standard for predicting survival in patients with mRCC but metastatic sites influence prognosis too. Indeed, gland metastasis, such as pancreatic metastasis, are related to more favorable prognostic features, long response to TTs and prolonged survival (18). Lung and nodes metastasis, instead, are related to higher complete response rate in patients treated with nivolumab–ipilimumab (19). Bone metastasis has been identified as an independent prognostic variable associated with poor survival in patients with mRCC (20) and brain metastasis seem to influence prognosis when they are more than 4 (21, 22). Even though nephrectomy has not been reported as a prognostic factor or in prognostic scores, patients who underwent nephrectomy are usually patients with metachronous disease or more indolent disease, compared to patients with synchronous disease.

The first evidence regarding the role of nephrectomy in mRCC refers to patients treated with cytokines (IL-2, IFN-a). CN demonstrated improved survival rate in them (8, 23). Later, Heng et al. retrospectively reported data from IMDC. CN was related to OS and PFS benefit in patients treated with TT, compared to patients who did not it. Patients with four or more IMDC prognostic criteria did not benefit from CN, as well as patients with reduced life expectancy (24).

Recently, the phase III CARMENA trial has investigated the role of immediate CN followed by sunitinib versus sunitinib alone and the phase III SURTIME trial has compared immediate CN followed by sunitinib therapy, versus treatment with three cycles of sunitinib followed by CN. Results from both trials have showed that patients with intermediate and poor risk, according to MSKCC and IMDC criteria, should be more appropriately treated with systemic therapy, deferring upfront CN, whereas CN might be considered in good risk patients with low burden disease (9, 10).

Retrospective data from 1,541 patients included in an international, multicenter, prospective database, confirm that deferred CN in mRCC patients treated with upfront sunitinib is associated with improved OS, in appropriately selected patients, whereas upfront CN followed by sunitinib is associated to a lower probability of OS. Authors suggest that initial course of systemic treatment might be a way to identify patients with more aggressive biology, already destined to low survival and who consequently would not benefit from CN (25).

Nowadays, it is difficult to put into practice data about CN, because the influence on outcome of previous nephrectomy in mRCC patients treated with IO is not well defined.

Most of the patients included in the CHECKMATE025 (88%) underwent nephrectomy, so that a subgroup analysis was not performed and data about outcome of patients treated with IO stratified by nephrectomy are not available (11, 12).

Nevertheless, CN after receipt of IO currently remains limited to case reports (26–29), whereas results from CHECKMATE214 showed OS benefit for patients, who had previous nephrectomy (CN or RN), receiving Nivolumab–Ipilimumab versus Sunitinib.

Recently, update results from Javelin Renal 101 favor avelumab plus axitinib over sunitinib across prespecified subgroups, including prior nephrectomy (30). Post hoc analysis of the same trial showed that almost 20% of patients who did not undergo prior nephrectomy and 34.5% of patients, treated with avelumab–axitinib, had 30% or greater shrinkage of primary renal tumor from baseline compared to 9.7% in sunitinib arm (31). These results demonstrate that IO-Tyrosine Kinase Inhibitors (TKIs) combination is active on primary disease, even if in a small number of patients.

The above-mentioned reports seem to suggest an interplay between IO, immune system and primary tumor that need to be researched further.

Our study reported difference in IO-OS between patients who had previous nephrectomy and patients who did not it. Previous nephrectomy seems to extend OS in mRCC patients treated with immunotherapy. Analyzing patients with synchronous metastatic disease, who underwent CN, the benefit in OS was confirmed, compared to patients who did not have CN. This result confirms the findings of Bakouny et al. reported at the ASCO GU 2020. The authors, in a propensity score-based analysis, found that CN was associated with a significant OS benefit in patients treated with IO and TT both. 198 patients treated with IO were included in the analysis (32).

In our multivariate analysis, nephrectomy retained a significant association with OS irrespective of the gland metastases and IMDC score.

Reported PFS’ results do not demonstrate difference between patients who underwent nephrectomy and patients who did not it (4.8 *vs* 3.7 *p* 0.186). This result confirms that PFS is not a surrogate for OS in patients treated with IO and confirms the delayed benefit in PFS with nivolumab, as previously reported in CHECKMATE025.

Furthermore, our study demonstrates that gland metastases are related to better prognosis and outcome, as demonstrated in univariate and multivariate analysis. Biological and immunological effects of the primary tumor on IO, which are mostly unknown, might explain the different outcome between patients who underwent nephrectomy and patients who did not it.

Previous report from Wald et al. analyzed how RN could influence immune response, collecting the immune signature in subjects with RCC before and after nephrectomy. Authors reported that the removal of the tumor produced few changes in the cellular immune response at 1 month post-nephrectomy, for example the level of circulating BTLA(B and T lymphocyte attenuator)-expressing CD8+ T cells decreased significantly, suggesting a reversal of T-cell exhaustion and dysfunction (15).

Finally, it is noteworthy to mention the retrospective study by Pignot et al. regarding patients who underwent delayed nephrectomy following IO. Patients who received IO and who experienced complete response on metastatic sites, underwent nephrectomy to achieve complete response. At a median follow up of 15 months, 73% of patients were free from progression, but inflammatory infiltration after long exposure to IO resulted in challenging surgery (33).

Our study covered a well-balanced population and represented all risk categories according to IMDC (**Table 4**).

**Table 4:** Differences between patients who had nephrectomy *vs* patients who had not nephrectomy.

	Nephrectomy246 (85.7%)	No Nephrecomy41 (14.3%)
**Histology**
Clear cell	218 (88.6)	37 (90.2)
Non-clear cell	28 (11.4)	4 (9.8)
**Sarcomatoid component**
Y	34 (13.8)	2 (4.8)
N	212 (86.2)	39 (95.2)
**Bone metastasis**		
Y	67 (27.0)	17 (41.4)
N	182 (73.0)	24 (58.6)
**Lynphonodes metastasis**		
Y	105 (46.7)	23 (56.0)
N	141 (57.3)	18 (44.0)
**Lung metastasis**		
Y	102 (41.4)	20 (48.7)
N	144 (58.6)	21 (51.3)
**Liver metastasis**		
Y	25 (10.1)	8 (19.5)
N	221 (89.9)	33 (80.5)
**Gland metastasis**		
Y	29 (11.7)	3 (7.3)
N	217 (88.3)	38 (82.7)
**IMDC SCORE**		
0	80 (32.5)	2 (4.8)
1	143 (58.1)	33 (80.5)
2	23 (9.3)	6 (14.7)
**ECOG PS**		
0	128 (52.0)	17 (41.5)
1	96 (39.0)	20 (48.8)
2	22 (9.0)	4 (9.7)
**IO line**		
2	164 (66.6)	31 (75.6)
3	65 (26.4)	8 (19.6)
Further line	17 (7.0)	2 (4.8)

Y, Yes; N, No; mOS, median Overall Survival; IO, Immune-Oncology; IMDC, International

Metastatic Renal Cell Carcinoma Database Consortium; ECOG PS, Eastern Cooperative Oncology Group Performance Status; G, Grade.

Specifically, it included 30% of patients with bone metastasis, already known poor prognostic factor in mRCC, which is consistent with frequency of bone metastasis in RCC.

We analyzed patients who received IO mostly as second or third therapy, to homogenize our population and minimize the selection bias between patients with metachronous disease, or patients with low burden disease referred to CN, and patients with synchronous disease or with higher burden of disease referred to upfront systemic therapy. Indeed, recently, Donskov et al. demonstrated that, in patients treated with first line TKI, synchronous disease was associated with poorer OS and shorter Time to Treatment Failure (TTF) (34).

The purpose of our trial was to suggest that the persistence of primary renal cell tumor could influence the efficacy of Immunotherapy because it could influence the immune system. This is the reason why patients who had CN or RN were not divided into two groups.

In spite of the novel treatments available for renal cell carcinoma, our paper remains relevant because TKI monotherapy can still be considered as a standard of care for many patients. Indeed, IO-TKI failed to show an OS advantage over sunitinib in favorable risk patients according to IMDC and MSKCC score. Moreover, we included patients treated with IO as second line treatment since in Europe IO and IO-TKI has not been available for long time.

Limits of our study include the retrospective collection of data, the small sample size and the lack of central radiological review. As basis for further considerations, there is significant residual confounding in the analysis of nephrectomy versus no nephrectomy, particularly in the metachronous group. Those patients, who are not offered curative intent surgery, are likely more unwell and less fit and thus, perhaps, destined to do poorly regardless of type of systemic therapy received at the time of metastatic diagnosis.

Furthermore, patients who had nephrectomy were more likely to be favorable risk and this might bias results of multivariate analysis.

Our assumption can be made that resection of primary tumor could have an effect on immune system and IO response, even if IO treatment is administered long after surgical intervention. Data are still unclear and further prospective trials would assess this issue.

## Conclusion

In our real-world experience in mRCC patients treated with IO, previous nephrectomy, including CN, seems to be associated with a better outcome, in terms of OS, with all the limitations of a retrospective collection. The benefit of previous nephrectomy persisted also in multivariate analysis.

## Data Availability Statement

The raw data supporting the conclusions of this article will be made available by the authors, without undue reservation.

## Ethics Statement

The studies involving human participants were reviewed and approved by the Campus Bio-Medico University of Rome. The patients/participants provided their written informed consent to participate in this study.

## Author Contributions

MSt and DS provided study conception and design. GP made critical revisions. All authors contributed to the article and approved the submitted version.

## Conflict of Interest

The authors declare that the research was conducted in the absence of any commercial or financial relationships that could be construed as a potential conflict of interest.
