# Supplementary figures and images for: Impact of Previous Nephrectomy on Clinical Outcome of Metastatic Renal Carcinoma Treated With Immune-Oncology: A Real-World Study on Behalf of Meet-URO Group (MeetUro-7b)
Source: Front Oncol. 2021 Jun 8;11:682449. doi: 10.3389/fonc.2021.682449 (PMC8217989; doi:10.3389/fonc.2021.682449)

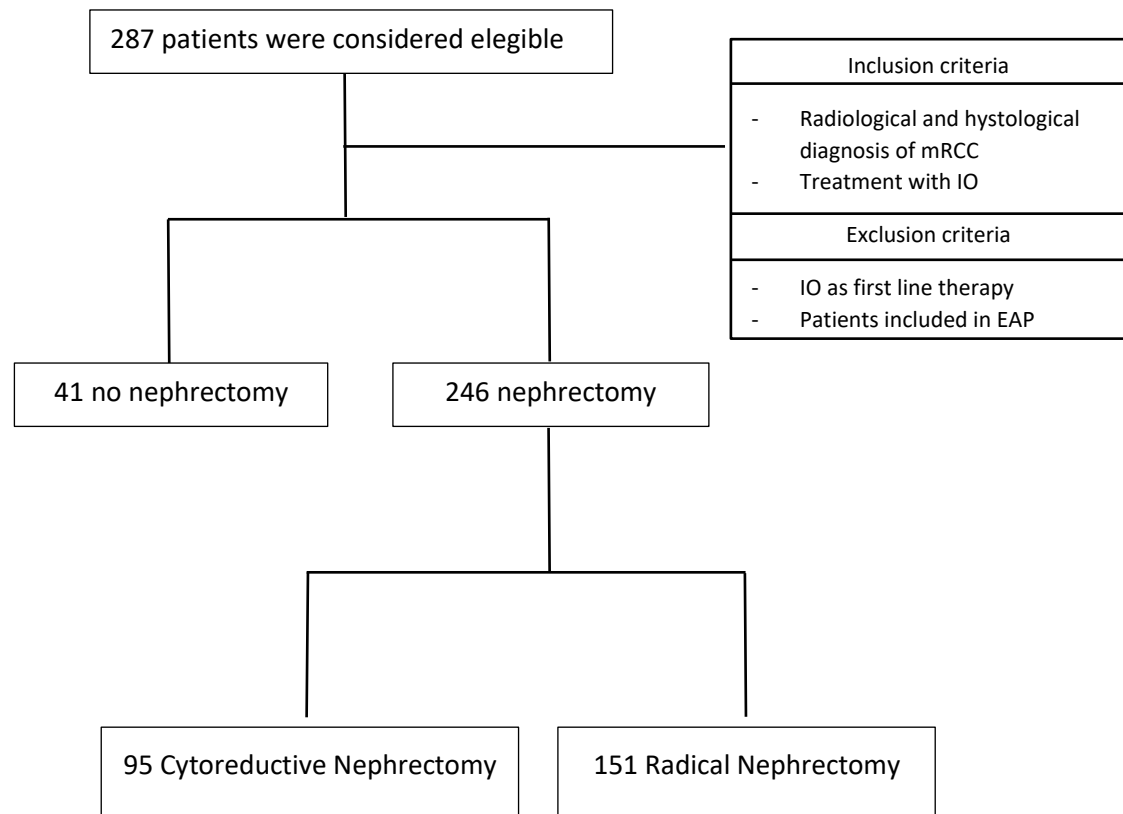

Supplement: Supplementary file 1 [file DataSheet_1.pdf]
